# Paper-Based Aptasensor Assay for Detection of Food Adulterant Sildenafil

**DOI:** 10.3390/bios14120620

**Published:** 2024-12-17

**Authors:** Murat Kavruk, Veli Cengiz Ozalp

**Affiliations:** 1 Department of Medical Biology, School of Medicine, Istanbul Aydin University, Istanbul 34292, Turkey; muratkavruk@aydin.edu.tr; 2 Department of Medical Biology, School of Medicine, Atilim University, Ankara 06830, Turkey

**Keywords:** adulteration, sildenafil, biosensors, lateral flow strip assay, mesoporous silica nanoparticles

## Abstract

Sildenafil is used to treat erectile dysfunction and pulmonary arterial hypertension but is often illicitly added to energy drinks and chocolates. This study introduces a lateral flow strip test using aptamers specific to sildenafil for detecting its illegal presence in food. The process involved using graphene oxide SELEX to identify high-affinity aptamers, which were then converted into molecular gate structures on mesoporous silica nanoparticles, creating a unique signaling system. This system was integrated into lateral flow chromatography strips and tested on buffers and chocolate samples containing sildenafil. The method simplifies the lateral flow assay (LFA) for small molecules and provides a tool for signal amplification. The detection limit for these strips was found to be 68.2 nM (31.8 µg/kg) in spiked food samples.

## 1. Introduction

Fast detection of illegal adulteration with chemical drugs is a significant challenge in the food safety field. Illicit food additives are substances that are either banned or not approved for use in food products due to their potential health risks. These additives are sometimes used illegally by manufacturers to cut costs or enhance certain food qualities, but they can pose serious health hazards to consumers. Sildenafil, the active ingredient for treating erectile dysfunction, is not legally permitted as a food additive. However, it has been found illicitly in some dietary supplements, energy beverages, and chocolate products. Sildenafil or its derivatives were identified in 65% (*n* = 49) of tested food products in Croatia and 56.3% (*n* = 32) in Saudi Arabia [1,2]. The Food and Drug Administration (FDA) database contains tainted products advertised as dietary products that were adulterated with hidden drugs. The predominant category of these tainted products was phosphodiesterase inhibitors, including sildenafil derivatives [3]. These supplements are often marketed as natural or herbal products but contain undeclared pharmaceutical ingredients, posing significant health risks to consumers.

Several analytical methods are used for the determination of sildenafil in food samples, particularly in cases of adulteration in food products. These approaches range in complexity, sensitivity, and specificity. High-performance liquid chromatography (HPLC) is a commonly used sildenafil detection method due to its dependability, precision, and capacity to handle complicated matrices like dietary supplements. HPLC-MS or UV detection are widely used as detection techniques coupled with HPLC systems [4]. LC-MS/MS is another sensitive and specific approach for detecting sildenafil in food. It outperforms HPLC in accuracy and precision. Gas chromatography–mass spectrometry (GC-MS) is another approach with good specificity and sensitivity for complicated food matrices. It handles volatile substances well, making it acceptable for contaminated products [5]. Thin-layer chromatography (TLC) is a cheaper and simpler way to test food samples for sildenafil. Surface-enhanced Raman spectroscopy (SERS) can detect trace sildenafil levels in a short time duration [6]. Electrochemistry- or fluorescence-based methods have been reported for fast determination of sildenafil [7,8,9].

Lateral flow test strips stand out as one of the most convenient formats for fast-responding biosensors, primarily due to their simplicity and cost-effectiveness [10]. These devices have found widespread success in various applications, most notably in home testing kits, such as pregnancy tests and glucose monitoring strips for diabetes management [11]. These applications rely on antibody capture or enzymatic signaling mechanisms, which enhance their reliability and accuracy. In addition to these well-known uses, lateral flow immuno-strips have emerged as valuable tools in many other medical diagnostics. They facilitate the rapid detection of specific diseases and conditions, such as the identification of *Helicobacter pylori* in patients with gastritis or the detection of H1N1 virus in cases of suspected influenza [12]. These immunoassays provide the specificity of antibodies to provide quick and actionable results, making them essential in clinical settings. A recent study reported paper-based colorimetric detection of sildenafil in functional food capsules based on nanomaterial interaction with a 58 ng/mL detection limit [13]. However, the development of lateral flow strips for the detection of small molecules presents significant challenges, particularly when these molecules are not enzymatic substrates. The complexity of creating effective recognition elements for small non-enzymatic molecules often limits the feasibility of such tests. As a result, while enzymatic substrates can be easily integrated into lateral flow assays, the same cannot be said for a broader range of small analytes, which require more sophisticated detection strategies. This limitation highlights the ongoing need for innovation in the design and functionality of lateral flow biosensors, particularly for applications beyond traditional enzymatic or specific nanomaterial-based signaling.

Nucleic acid aptamers can bind target molecules with high affinity and high specificity. Aptamers are in vitro selected, potentially, for any given target, through a combinatorial selection process known as systematic evolution of ligands by exponential enrichment (SELEX). Once they are identified specifically for the target, aptamers can be synthesized in high purity and at a relatively low cost, meaning no batch-to-batch variability [14]. Proteins, peptides, small molecules, or whole cells can be a target for aptamer binding. Their specificity is associated with the unique secondary and tertiary structures that each sequence can assume, making them highly sensitive to the structure of their targets. The completely in vitro selection of aptamers is one of the major advantages, compared with antibodies that are produced through biological systems. Moreover, chemical synthesis of aptamers provides a cost-effective and reliable source of affinity molecules. Aptamers have been proposed for the possible replacement of antibodies in different formats of bio-affinity assays, demonstrating their potential for delivering real-time, rapid-acting sensing of small molecules from a variety of sample types [14,15].

Aptamer technology has provided an efficient route to point-of-care device development when combined with a lateral flow assay (LFA) biosensor platform [16]. Recently, a number of aptamer-based LFAs have been reported for small molecule targets. Sandwich LFAs and competition LFAs are the most common types of LFA. When there are two target-specific aptamers available, as is the case when detecting high-molecular-weight analytes with several specific binding sites, the sandwich approach is favored. When there is just one aptamer specific to a target, or when the molecular weight of the analyte being detected is very low, the competition approach is utilized for detection. For small molecules, displacement-based aptamer sensors have been utilized as the detection principle for some analytes [17]. The hybridization chain reaction method has been utilized to amplify the signal produced by displacement-based systems [18]. The recent progress in aptamer technology provides an opportunity to apply the potential of the aptamer and LFA to building a promising platform for highly efficient point-of-care device development.

**Figure 1:**
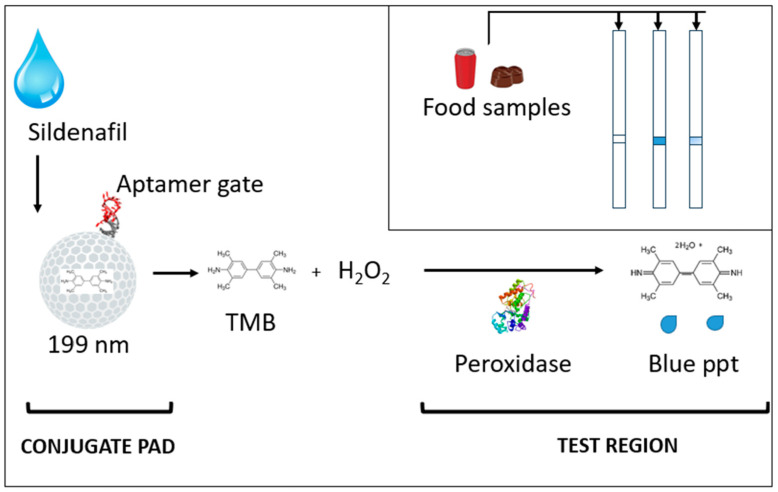
The principle of sensing strategy for sildenafil-containing samples.

The main objective was to investigate a novel approach to overcome one of the major challenges in strip biosensing of small molecules. This approach depends on aptamer recognition-based signal production. Thus, it can potentially be applied to any small molecules since specific aptamers can be obtained against any small molecules. The mesoporous silica nanoparticles (MSNP) were loaded with a reporter molecule (TMB), and the pores were capped by aptamer molecules. The aptamer-capped MSNPs were then placed in the conjugate pad region of a lateral flow strip, which has a test region prepared by adsorption of horse radish peroxidase (HRP). Figure 1 shows that the application of sildenafil-containing samples onto sample region of a strip causes the release of entrapped TMB molecules upon molecular interaction between aptamer and its target sildenafil. The released TMB travels along the strip to reach the test region with immobilized HRP, which produces a visible blue precipitate to reflect the concentration of sildenafil in the food sample.

## 2. Materials and Methods

### 2.1. Materials

The chemical compounds were acquired from Sigma-Aldrich (Atasehir, Turkiye). The SELEX oligonucleotides were designed as reported previously and manufactured by Oligomers Ltd. (Ankara, Turkey) (Table 1). The SELEX library had a total length of 75 nucleotides, comprising a central segment containing 40 randomly generated nucleotide sequences, along with fixed primer sites positioned at both ends to facilitate PCR reactions. Graphene oxide (GO) and other chemicals were obtained from Sigma and used as they are.

### 2.2. Aptamer Selection

Sildenafil aptamers were selected by following GO-SELEX as described previously [19]. The details of the selection procedure can be found in the Supplementary Information. The DNA library and PCR primers were according to published reports. SELEX cycles were performed by mixing 50 nmol library with 100 nmol sildenafil molecules in PBS buffer (0.01 M phosphate buffer, 0.0027 M potassium chloride, and 0.137 M sodium chloride, pH 7.4) and incubating for 30 min. at 25 °C. Subsequently, 4 mg/mL GO was added and further incubated for 30 min. under same conditions. The unbound DNA library members interacted with GO and were collected by centrifugation at 10,000× *g* for 10 min. The supernatant that contained sildenafil-bound oligonucleotides was collected and amplified by PCR.

Casein in PBS (1%) was used in the negative selection rounds to eliminate common binders of the SELEX library since it is the main component in chocolate. In the subsequent stages, aptamer candidate sequences were determined in the enriched SELEX cycle by next generation sequencing by using NGS primers (Table 1) and analyzed with bioinformatics methods. In the final stage, the oligonucleotides whose sequences were obtained and characterized using a fluorescent binding assay in affinity experiments performed after fluorescently labelled aptamer candidate sequences, and their binding affinities (Kd) were calculated.

### 2.3. Synthesis of Aptamer-Gated Mesoporous Silica Nanoparticles

Mesoporous silica nanoparticles (MSNP) were manufactured using the methods described previously [20]. A solution of N-cetyltrimethylammonium bromide (CTAB) was combined with NaOH and kept at a temperature of 80 °C while being stirred continuously. Tetraethoxysilane (TEOS) was slowly added in drops over a period of 24 h throughout the duration of the reaction. The MSNPs were collected on a filter as a white precipitate and washed with water and methanol. The CTAB template was eliminated using HCl refluxing for generating pores on the surface of the particles. Ultimately, nanoparticles were acquired by the process of filtration followed by drying. The dry particles were analyzed using transmission electron microscopy (TEM). The hydrodynamic diameter of MSNPs was determined using dynamic light scattering (DLS) analysis. Epoxy-functionalized MSNPs were synthesized by the addition of (3-Glycidyloxypropyl)triethoxysilane (GOPTES), followed by overnight mixing. The details of the attachment procedure are provided in the Supplementary Information.

Following three washes with 1X PBS, thiol-labelled sildenafil-binding aptamers were combined under slightly alkaline conditions at room temperature and allowed to covalently connect overnight. The epoxy grafting quantity was evaluated using a titration method based on pyridine-HCL, as previously published [21].

The trimethyl benzidine (TMB) was loaded into the mesopores of the MSNPs through a straightforward process of incubating them overnight. The aptamers, which were labeled with amines, were attached to the particles using epoxy linking reaction in a carbonic acid buffer with a pH of 9.4, following a process that was previously published [22]. The TMB loading was performed using a peroxidase assay. Aptamer gates on the surface of silica nanoparticles were quantified using spectrophotometric measurements at a wavelength of 260 nm. The TMB-loaded aptamer-gated silica nanoparticles (Apt-SiNp@TMB) were dried and kept refrigerated until use.

### 2.4. Strip Experiments

The lateral flow strip assays were performed according to the methodology outlined in previous reports [20,23]. The strips were assembled on cardboard by sequentially fixing the sample pad, conjugate pad (glass fiber), nitrocellulose membrane (nitrocellulose HF120), and absorbent pad. Horse radish peroxide (HRP) was immobilized on the test zone in the middle of the nitrocellulose part via physical adsorption by drop and dry method. The Apt-SiNp@TMB was immersed in the conjugate pad and subsequently dried prior to the construction of the strip. In each assay, a 100 μL sample in PBS was directly placed on the sample pad, and the images were captured after 5 min incubation at room temperature. The intensities of TMB precipitates in the test region were produced by peroxidase activity and quantified from images using ImageJ 1.46r [24].

### 2.5. Fluorescent Assay

Sildenafil or casein in PBS was mixed with fluorescently labelled SDF-Aptamer at 1 µM. The non-binders were removed by adding 4 mg/mL GO, incubating for 60 min, and performing centrifugation. The aptamer amount in the supernatant was quantified by spectrofluorimetric measurements.

### 2.6. Food Samples

In order to use chocolate as a matrix for sildenafil detection, 2 g of chocolate samples were obtained from local markets and crushed in a mortar in 10 mL methanol for 30 min [25]. An equal amount of commercially available beverage was mixed with homogenized chocolate sample. The solution was analyzed for the presence of sildenafil by following a spectrofluorimetric method with excitation at 340 nm and emission at 430 nm [26]. There was no detectable peak in the samples used in preparing sildenafil-spiked samples.

## 3. Results and Discussion

In this study, a methodology for lateral flow assays was developed for small molecule detection on site. Although lateral flow strip biosensors are highly desirable for small molecule targets (widespread in medicinal, environmental, food safety, and industrial applications), challenges originating from difficulty in molecular interaction should first be explored for designing better LFAs with stable signal production. Mostly, competitive assays are employed for small molecule strip assays, requiring extra labelling of target molecules, low sensitivity, and specificity. Here, we used an approach that can provide easy preparation strips for any small molecules for highly sensitive and specific biosensors. This approach was adapted from a previous successfully applied nanoparticle system for whole bacteria cells [20]. In this design, the interaction of signaling nanoparticles in the conjugation pad and resulting free signal molecule (TMB) migration and production of visible signal at test regions was achieved in a short time. An important food safety target, sildenafil was used as the target in this study.

### 3.1. Sildenafil Aptamer Selection

Sildenafil is water-insoluble and used in citrate salt form for improving its solubility. It is a selective and reversible inhibitor of phosphpodiesterase type 5 enzyme used for the treatment of erectile dysfunction and pulmonary arterial hypertension. Tadanafil and vardenafil are two common analogues used in medical applications. As active pharmacologic agents, usage of these substances in food is forbidden at an international level. However, there are various entries and publications about food adulteration via these molecules in different countries. Food fraud with sildenafil has become a worldwide issue recently. The RASFF (for the EU) and the FDA (for the USA) have database entries about the undeclared adulteration of food products with sildenafil and/or its chemical derivatives in which sildenafil is the dominant adulterant. Therefore, this study aimed to select an aptamer that can recognize sildenafil, especially for on-site analysis in surveillance and inspection activities.

**Figure 2:**
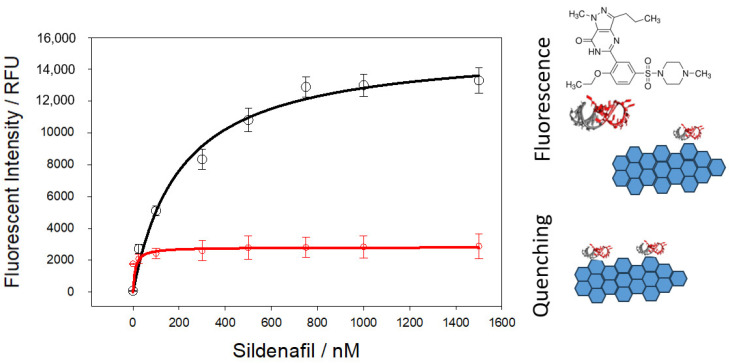
Fluorescent binding analysis of sildenafil-binding aptamer for sildenafil (black line) and casein (red line). The assay is based on the detachment of FAM-labelled aptamers from GO surface by sildenafil molecules.

In this study, sildenafil was used to select a specific aptamer sequence by following the graphene oxide systematic evolution of ligands by exponential enrichment (GO-SELEX) procedure. In each round, ssDNA library members were mixed with the target molecule sildenafil in PBS. Negative selection was performed after round 3 by using chocolate matrix. About 2.6-fold enrichment was achieved in 11 rounds (Figure S1). The final ssDNA pool (round 14) was sequenced by next generation sequencing. First, index, adaptor, and 5′ and 3′ constant primer regions were filtered for correct insert size (40 bp random region). Paired end reads were merged to obtain 14.567 reads. One of the motifs (ACTCG) was shared by 1013 sequences (7% of the total sequences). Therefore, one of the sequences (5′-ATTGCAAACCCTCTACCAACAATCGTACTCGTTCGACGTGA) with this motif was characterized for binding affinity to sildenafil (SDF-Aptamer). The affinity constant (K_D_) for the aptamer was estimated as 205.3 ± 38.1 nM by a fluorescent assay from Langmuir one-ligand binding equation fitting to sildenafil concentration [27]. For specificity, casein was used since it is one of the main ingredients of chocolate and frequently used in Western blot analysis for reducing background binding of nucleic acids on nitrocellulose membranes. Non-specific binding of the aptamer to casein at various concentrations was negligible compared to sildenafil binding (Figure 2).

The sildenafil aptamer selected in this work exhibits optimum binding affinity in PBS buffer at 25 °C. It is known that extreme pH, ionic strength, or temperature can affect aptamer performance. For example, the thrombin aptamer was protonated at three critical nucleotides under low pH conditions, resulting in a reduction in its binding efficiency. Similarly, the cadmium aptamer demonstrated reduced binding efficiency under such conditions [28].

### 3.2. Aptamer Gated SiNP@TMB

**Figure 3:**
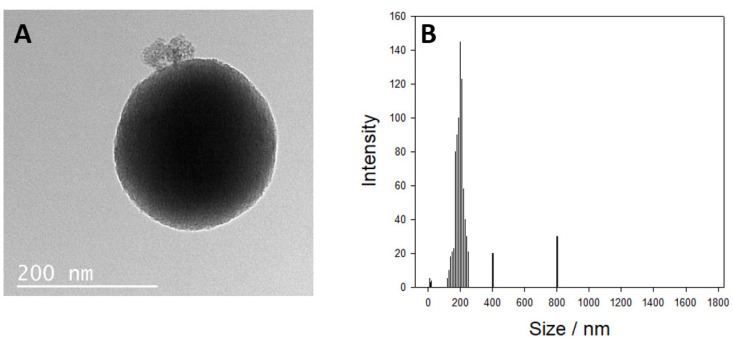
The mesoporous silica particles were analyzed by (**A**) TEM for morphology and (**B**) DLS for size distribution.

The signaling system for sildenafil is based on an aptamer gate structure, which can respond to the presence of sildenafil molecules by releasing a reporter molecule, TMB. The selected aptamer sequence was converted to a molecular gate structure by adding 6-base sequences at the 3′ end (TGCAAT), which is complementary to the 5′ end of the molecule, to form a hairpin structure. Similar signaling systems have been reported in various applications. Nanoparticles possess a very large surface area and, most significantly, mesoporous silica has a homogeneous and controlled porous structure that exhibits good loading capacity. Mesoporous silica nanoparticles have the capacity to effectively transport concentrated report molecules for targeted sensing, using capping molecules like aptamers, oligonucleotides, and antibodies [29,30]. The silica nanoparticles were synthesized by following published protocols, and 199 ± 4 nm in size, round-morphology nanoparticles were obtained (Figure 3). BET analysis demonstrated that area and mean pore volume were 677.32 m^2^/g and 0.352 cm^3^/g, respectively. The SiNP was grafted with epoxy groups by a salinization procedure with GOPTES and covalently conjugated to 5′-amine groups of aptamer molecules.

**Figure 4:**
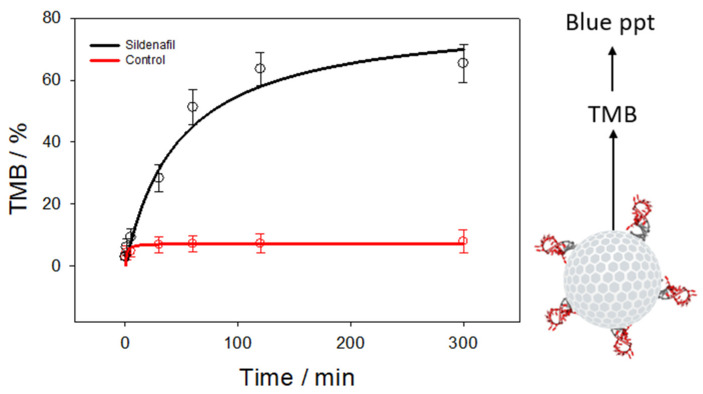
The release graphics of TMB molecules from aptamer-gated silica nanoparticles in the absence of (control) or in the presence of 1 µM sildenafil. The interaction of sildenafil with aptamer gates releases TMB cargo, which is oxidized to form blue-colored precipitate.

The proper operation of the aptamer-capped signaling system of a sensor device necessitates the prevention of trapped guest molecules from escaping when the aptamer gate molecules are closed as intended. The capping procedure involved immobilizing approximately 18.3 ± 1.1 pmoles of aptamers per milligram of nanoparticles, and it successfully trapped 16.4 ± 2.2 picomoles of TMB molecules. A minor leakage of (7.2 ± 4.4%) (*n* = 3; average of three independent experiments) of the maximum loading was seen in 2 h (Figure 4). This level of leakage may be considered acceptable compared to similar previous studies. However, TMB release increased rapidly for the first 2 h. Then, the TMB release signal increased further to reach a plateau at around 63 ± 6.2% of the total TMB. Previous studies with fluorescein or TMB as reporter molecules gave similar values for their target molecule ATP [31,32]. These results showed that Apt-SiNP@TMB particles are ready for strip development for sildenafil determination.

### 3.3. Sildenafil AptaLFA

**Figure 5:**
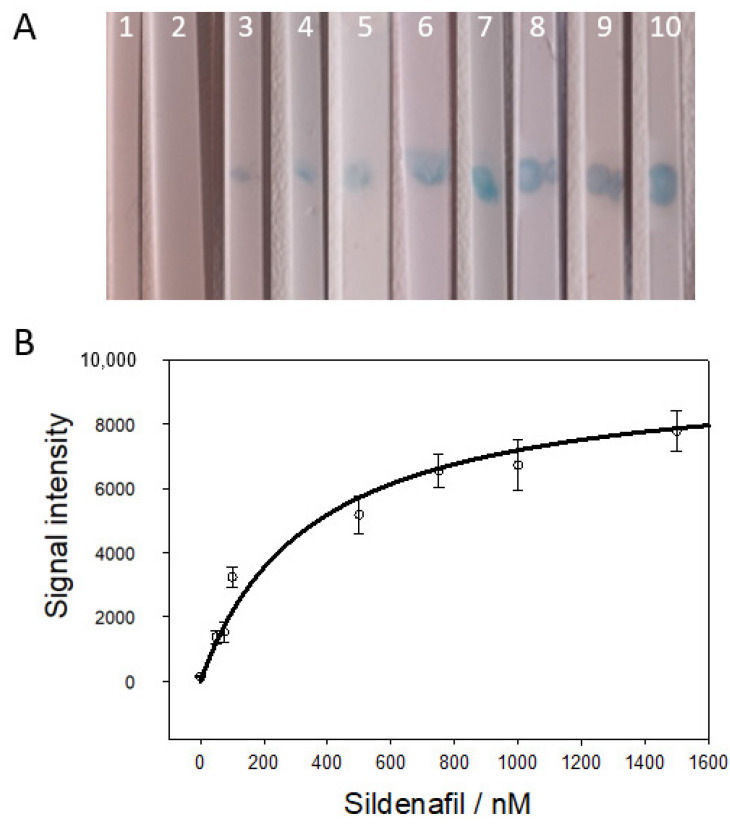
Sildenafil test strips used for spiked chocolate samples. (**A**) Strip test results when sildenafil samples were added. (Strip 1 is no Apt-SiNP@TMB strips and 1500 nM sildenafil sample, strips 2–10 include Apt-SiNP@TMB in the conjugate pad with the sildenafil-containing samples; strip 2: 0 nM, strip 3: 5 nM, strip 4: 50 nM, strip 5: 100 nM, strip 6: 250 nM, strip 7: 500 nM, strip 8: 750 nM, strip 9: 1000 nM, strip 10: 1500 nM.) The strip images were captured 5 min after the sample was applied. (**B**) The band intensity of LFA strips. The error bars represent the standard deviation of triplicate experiments.

We investigated Apt-SiNP@TMB in the strip conjugate pad region for specific determination of sildenafil-spiked chocolate samples. Sildenafil aptamer strips were used for spiked chocolate samples for sildenafil determination (Figure 5). Samples containing 1–1500 nM sildenafil resulted in visible blue bands in 5 min assay time (Figure 5A, strips 2–9). The TMB signal was calculated from the intensity of the bands on the strips shown in Figure 5A, as presented in Figure 5B. Apt-SiNP@TMB at the conjugate pad region and sildenafil molecules in the sample causes the release of TMB, which migrates to the test region with the capillary flow and leads to chemical conversion to blue precipitate by HRP, which can be visually identified or quantified with regard to intensity. The blue bands of strips 6, 7, 8, and 9 (500 nM and higher concentrations of sildenafil) cannot be distinctly identified by visual inspection. Similarly, strips 2 and 3 or strips 4 and 5 could not be distinguished visually, but they could be quantified by intensity analysis. Thus, visual inspection can be used for qualitative analysis for the presence of sildenafil in the samples up to 500 nM. A linear relationship between TMB release and sildenafil concentration was obtained between 100 nM and 750 nM with a high correlation (R2 = 0.98). The limit of detection (LOD) was 68.2 nM. Other types of sildenafil detection methods reported a wide range of LOD for sildenafil, between 21 nM to 100 µM [33,34]. A recent fluorescence-based immune-LFA achieved 0.08–1.66 ng mL^−1^ for sildenafil [35].

The aptamer-based paper assay approach in this study presents detection advantages for sildenafil over traditional methods such as liquid chromatography–mass spectrometry/mass spectrometry (LC-MS/MS) and high-performance liquid chromatography (HPLC). Although the sensitivity of our method is less compared to these traditional methods, portability and detection speed at low cost are important key features. The detection time in paper-based sildenafil detection is within minutes, and the biosensor is highly portable. In general, colorimetric LFAs have significant potential for commercialization due to their simplicity, cost-effectiveness, and fast detection ability. LFAs are low-cost tests, making them accessible for large-scale applications. In fact, the reagents used in this study (specifically silica nanoparticles and aptamers) can be easily incorporated in the strips without changing the final price much. Traditionally, LFAs have lower sensitivity compared to laboratory-based techniques. However, the signal application capacity of aptamer-gated signaling can be optimized to achieve the limit of detections required for sildenafil adulteration detection. LFAs are known for having a long shelf life without any need for special conditions like refrigeration for storage [36]. To investigate the shelf-life of the strips, we tested the strips after storing them at room temperature for up to 3 months. The signal intensity for the same concentration sample demonstrated stable outcome at the same level for the first month (Figure S3). The signal decreased slightly for the second and third months (about 7.2% of the average values).

## 4. Conclusions

Sildenafil is a pharmacologically active chemical whose pharmacodynamics are a driving force for illegal food adulteration. Although there are laboratory-based analysis methods, an on-site solution for inspection and surveillance of food products against sildenafil is yet to be solidified or commercialized. We demonstrated a colorimetric qualitative test strip for sildenafil, which can be further studied for quantitative determination. From this perspective, this study is a proof of concept for sildenafil detection with an aptamer-based lateral-flow sensor.

## Figures and Tables

**Table 1:** The oligonucleotide sequences used in this study.

Name	Sequence
SELEX Library	5′-GGCGGCGATGAGGATGAC-N_40_-ACCACTGCGTGACTGCC-3′
Forward primer	5′-GGCGGCGATGAGGATGAC-3′
Reverse primer	5′-GGCAGTCACGCAGTGGT-3′
Biotynilated primer	5′-Biotin- GGCGGCGATGAGGATGAC-3′
Fluorescein labelled primer	5′-Fluorescein- GGCGGCGATGAGGATGAC-3′
NGS-forward primer	5′-TCGTCGGCAGCGTCAGATGTGTATAAGAGACAGGGCGGCGATGAGGATGAC-3′
NGS-reverse primer	5′-GTCTCGTGGGCTCGGAGATGTGTATAAGAGACAGGGCAGTCACGCAGTGGT-3′

## Data Availability

Dataset available on request from the authors.

## Supplementary Materials

The following supporting information can be downloaded at https://www.mdpi.com/article/10.3390/bios14120620/s1, Methods for Aptamer selection, Aptamer attachment on the surface of nanoparticles, Shelf Life of the Strips; Figure S1: Enrichment; Figure S2: modification of mesoporous silica nanoparticles; Figure S3: Shelf life. References [37,38,39,40,41,42,43] are cited in the Supplementary Materials.
