# Supplementary material for: Paper-Based Aptasensor Assay for Detection of Food Adulterant Sildenafil
Source: Biosensors (Basel). 2024 Dec 17;14(12):620. doi: 10.3390/bios14120620 (PMC11674377; doi:10.3390/bios14120620)
Supplement: Supplementary file 1 [file biosensors-14-00620-s001.zip › biosensors-3342018-supplementary.pdf]

**Supplementary Information**

# Paper-based aptasensor assay for detection of food adulterant sildenafil

[1] Istanbul Aydin University, School of Medicine, Department of Medical Biology, Istanbul, Turkey; muratkavruk@aydin.edu.tr
[2] Atilim University, School of Medicine, Department of Medical Biology, Ankara, 06830 Turkey; cengiz.ozalp@atilim.edu.tr

## Aptamer selection

Sildenafil aptamers were selected by following graphene oxide GO-SELEX as described previously [1]. The procedure is based on the efficient separation of unbound library oligonucleotides GO The DNA library and PCR primers were according to published reports as given in Table 1 [2,3]. SELEX cycles were performed by mixing 50 nmol library with 100 nmol sildenafil molecules in one mL of PBS buffer (0.01 M phosphate buffer, 0.0027 M potassium chloride and 0.137 M sodium chloride, pH 7.4) and incubating for 30 min. at 25 °C. Subsequently, 4 mg/mL GO was added and further incubated for 30 min. under same conditions. The unbound DNA library members interacted with GO and collected centrifugation at 10,000 x g for 10 min. The supernatant that contained sildenafil bound oligonucleotides was collected and amplified by PCR. The PCR conditions was optimized for each SELEX rounds for each PCR cycle to obtain the correct molecular weight band in a 4% agarose gel electrophoresis analysis (2 min at 95 °C, optimized cycles between 8–21 cycles of 30 s at 95 °C, 30 s at 55 °C, 30 s at 72 °C, and finally 5 min at 72 °C). The confirmed PCR products were amplified at large quantities, and a streptavidin-biotin procedure was applied for preparing ssDNA of library for the next round of SELEX. Streptavidin functionalized magnetic microparticles (100 μL) were mixed with the PCR products and incubated for 60 min. at room temperature by continuous mixing, capturing 3′-biotin-labelled dsDNA oligos. The mixture was washed with PBS three times and solubilized in distilled water. The unlabeled strands were collected from supernatant magnetically at 90 °C.

Casein in PBS (1 %) was used in the negative selection rounds to eliminate common binders of the SELEX library since it is the main component in chocolate. We performed 14 cycles in this binding buffer. After each cycle, the binding amount of the library to sildenafil was quantified by labelling the library with fluorescein. The fluorescently labelled ssDNA was mixed with GO and then 100 μM sildenafil in the same conditions as SELEX rounds. The supernatant was collected after centrifugation and quantified for fluorescence signal (ex. 480 nm, emm. 520 nm) (Figure S1).

Aptamer candidate sequences were determined in the 14$^{th}$ rounds of enriched library by next generation sequencing by using NGS primers (Table 1) [4] and analyzed with bioinformatics methods. MEME Suite program STREME protocol was used to determine the sequences with common regions of sequences [5].

In the final stage, the oligonucleotides whose sequences were obtained and characterized using a fluorescent binding assay in affinity experiments performed after fluorescently labelled aptamer candidate sequences, and their binding affinities (Kd) were calculated.

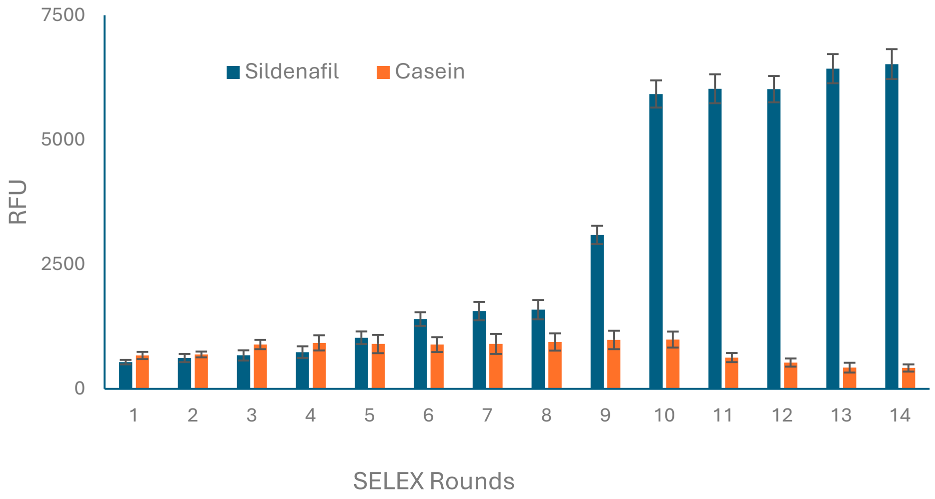


**Figure S1.** Enrichment of the amount of bound library members in each SELEX rounds.

**Aptamer attachment on the surface of nanoparticles**

The silica nanoparticles were synthesized by following published protocols [6]. Silica surfaces have been frequently silanized with non-selective covalent attachment of reactive silanes containing functional groups [7] Epoxysilanes were used to provide easy and strong attachment of biomolecules to glass surfaces, including silica [8]. The procedure was summarized in a schematic drawing (Figure S1). In reaction 1, silica nanoparticles (10 mg) were added into 10 mL of ethanol with 1 mM acidic acid and mixed at room temperature for 30 min. (3-Glycidyloxypropyl)triethoxysilane were added into this mixture at 2.5% and mixed for 6 hours. The epoxy modified particles were collected by centrifugation (14.000 rpm, 5 min) and washed three times with PBS buffer. In reaction 2, the 5'-amine labelled aptamer gate sequences (10 pmol) were mixed with epoxy-silica nanoparticles in basic carbonate buffer (100 mM sodium carbonate, pH=10.0) overnight. The excess epoxy groups were blocked by 50 mM ethanolamine.

**Shelf Life of the Strips**

The strips were kept in dry form at room temperature up to 3 months and tested with spiked samples (50 nM sildenafil). The sensor strips were prepared under the same conditions, air-dried and stored in a sealed bag purged with nitrogen gas at 25 °C until use.

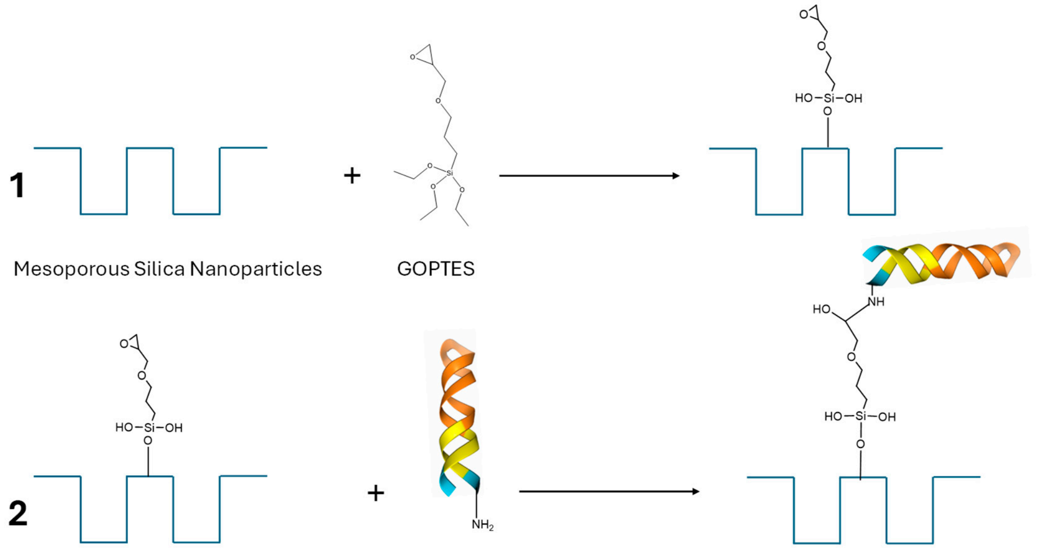


**Figure S2.** The modification of mesoporous silica nanoparticles. 1) Mesoporous silica nanoparticles were silanized by GOPTES for epoxy group functionalization, 2) Epoxy groups on the surface of particles were reacted with the primary amines on the 5'-end of aptamer gate oligonucleotides.

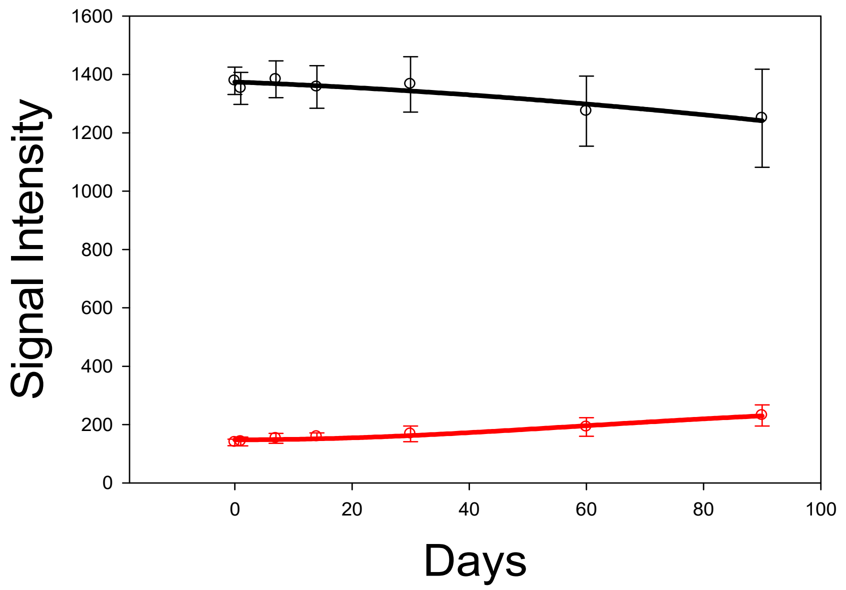


**Figure S3.** Shelf life determination for 90 days. The signals were calculated from images at 5 min. for Apt-SiNP@TMB at 50 nM sildenafil sample (Black line) and Apt-SiNP@TMB (Red line). Error bars indicate the standard errors of three independent experiments.

LFAs are known for long shelf life without any need for special conditions like refrigerator for storage [9]. To investigate the shelf-life of the strips, we tested the strips after storing at room temperature up to 3 months.

References


1. Guan, J.; He, K.; Gunasekaran, S. Selection of SsDNA Aptamer Using GO-SELEX and Development of DNA Nanostructure-Based Electrochemical Aptasensor for Penicillin. *Biosens Bioelectron X* **2022**, *12*, 100220, doi:https://doi.org/10.1016/j.biosx.2022.100220.
2. Dursun, A.D.; Dogan, S.; Kavruk, M.; Busra Tasbasi, B.; Sudagidan, M.; Deniz Yilmaz, M.; Yilmaz, B.; Ozalp, V.C.; Tuna, B.G. Surface Plasmon Resonance Aptasensor for Soluble ICAM-1 Protein in Blood Samples. *Analyst* **2022**, *147*, 1663–1668, doi:10.1039/d1an02332b.
3. Dursun, A.D.; Borsa, B.A.; Bayramoglu, G.; Arica, M.Y.; Ozalp, V.C. Surface Plasmon Resonance Aptasensor for Brucella Detection in Milk. *Talanta* **2022**, *239*, doi:10.1016/j.talanta.2021.123074.
4. Ersoy Omeroglu, E.; Sudagidan, M.; Yurt, M.N.Z.; Tasbasi, B.B.; Acar, E.E.; Ozalp, V.C. Microbial Community of Soda Lake Van as Obtained from Direct and Enriched Water, Sediment and Fish Samples. *Sci Rep* **2021**, *11*, doi:10.1038/s41598-021-97980-3.
5. Bailey, T.L. STREME: Accurate and Versatile Sequence Motif Discovery. *Bioinformatics* **2021**, *37*, 2834–2840, doi:10.1093/bioinformatics/btab203.
6. Tuna, B.G.; Durdabak, D.B.; Ercan, M.K.; Dogan, S.; Kavruk, M.; Dursun, A.D.; Tekol, S.D.; Celik, C.; Ozalp, V.C. Detection of Viruses by Probe-Gated Silica Nanoparticles Directly from Swab Samples. *Talanta* **2022**, *246*, 123429, doi:https://doi.org/10.1016/j.talanta.2022.123429.
7. Abraham Ulman *Inorganic Thin Films*; 1995;
8. Tsukruk, V. V.; Luzinov, I.; Julthongpiput, D. Sticky Molecular Surfaces: Epoxysilane Self-Assembled Monolayers. *Langmuir* **1999**, *15*, 3029–3032, doi:10.1021/la981632q.
9. Majdinasab, M.; Badea, M.; Marty, J.L. Aptamer-Based Lateral Flow Assays: Current Trends in Clinical Diagnostic Rapid Tests. *Pharmaceuticals* 2022, *15*.
